# Perceived Health Related Quality of Life Outcomes with Silicosis Patients

**Published:** 2017-09

**Authors:** Salah ABERKANE

**Affiliations:** Dept. of Psychology, Faculty of Human and Social Sciences, Khenchela University, Khenchela, Algeria

## Dear Editor-in-Chief

Silicosis is the oldest occupational lung disease caused by the inhalation of dust crystalline silica (1). There is no effective cure for this endemic disease and the principal help we could provide for patients is to extend their survival and improve their Health-Related Quality of Life (HRQoL) (2). Silicosis patients in Algeria are reported to be young and to have low economic level. Silicosis has become the greatest threat to workers’ occupational safety (Algerian stone carvers) considered as the most serious occupational disease worldwide. Dozens deaths cases of workers exposed to silica dust were recorded in recent years in Tizi Ouzou and Batna (Algeria) while the number of patients with silicosis in many previous statistics, obtained from medical sources, state that 50% of 1200 workers exposed to silica dust in the region of Batna are suffering from silicosis (3). This report explained that silicosis is possible to increase and be an important occupational lung disease in Algeria because these stone carvers engage in this risky behavior (through exposition to silica dust despite health problems outcomes). There were no previous studies on perceived silicosis, especially in the Arabic research focused on HRQoL among patients with silicosis (4, 5).

Wellness is influenced by the individuals’ decisions and choices relating to their behaviors. Although some of them behave in ways that promote HRQoL, others behave in ways that affect their health outcomes negatively. The concept of unrealistic optimism includes individuals ignoring and underestimating the risks of their behviors, which can lead them to experience health problems outcomes (6).

The aim of the present study was to evaluate the HRQoL in patients with silicosis through silicosis representations and unrealistic optimism. A single-sample, cross-sectional research design was used for this study. A convenience sample of 64 individuals (male patients aged between 21 and 71 (mean age, 33.75±8.49 yr) having silicosis and living in the region of TKOUT/ Batna (Algeria) between Sep 2014 to Sep 2015, was recruited.

The study was approved by the Ethics Committee of the university and an informed consent was taken from all subjects before the study.

**Fig. 1:**
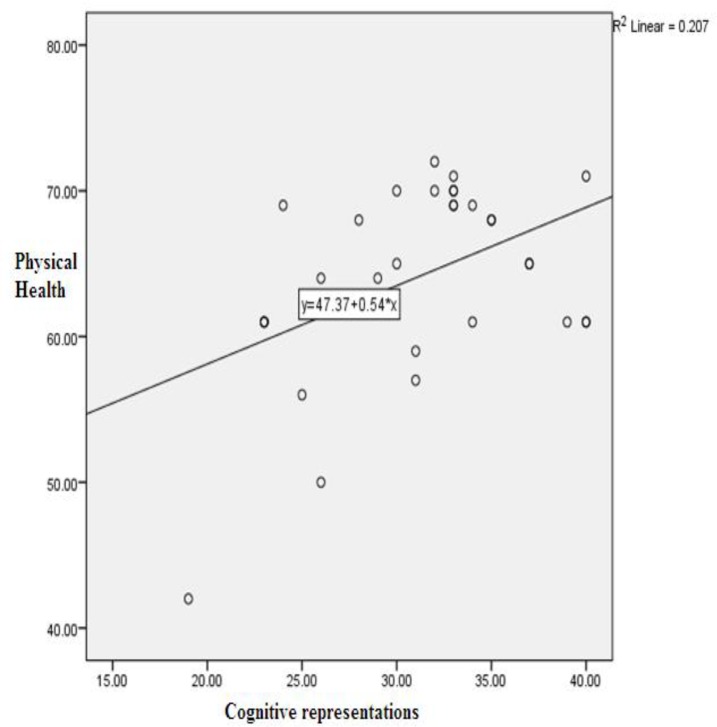
Impact of cognitive representations on physical health

**Fig. 2:**
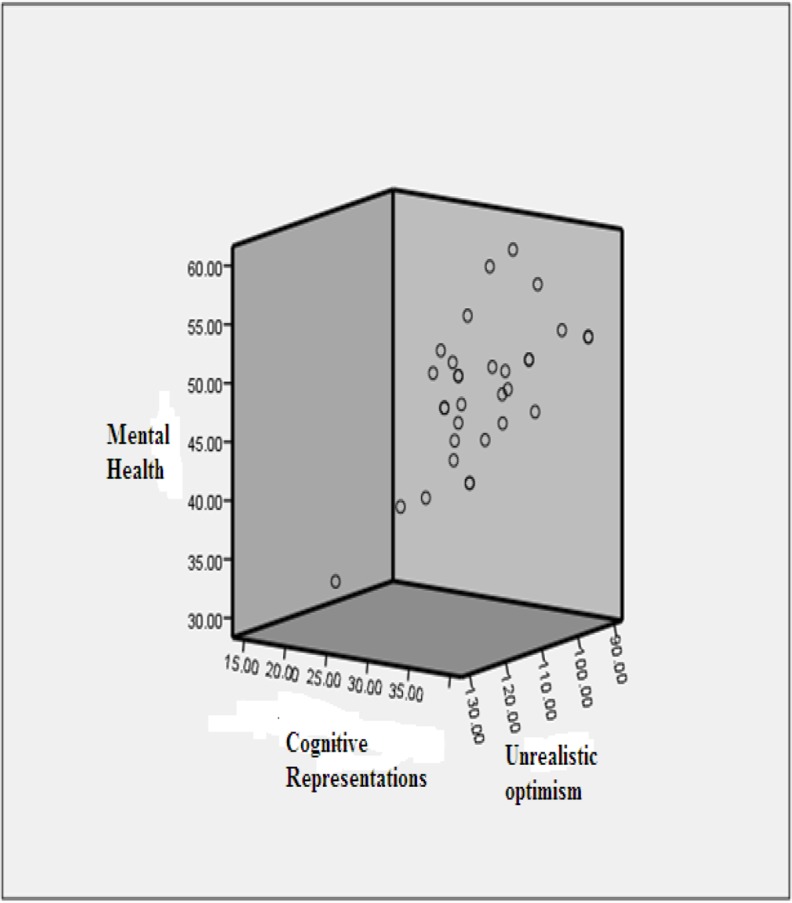
Impact of cognitive representations and unrealistic optimism on mental health

The mean length of total education was 1.72 ± 0.85 yr. Disease duration ranged between 8 months and 20 yr with a mean of 6.90 ± 4.33 yr. Data were collected using self-report information obtained from a Demographic Data Form, the Brief Illness Perception Questionnaire (B-IPQ), the Scale of Unrealistic Optimism, and the Medical Outcomes Study Short Form-36 ver. 2 (SF36v2). Significant correlations existed between cognitive representations, unrealistic optimism, and HRQoL. Cognitive representations are predictive of quality of life (dependent variables of mental health, and physical health). Cognitive representations with unrealistic optimism were better at predicting mental health (R2=0.30 & 0.44, respectively) than they were at predicting physical health (R2=0.21) (Fig. 1 and 2).

All regression equations had substantial residuals, indicating that accurate more prediction of the dependent variables was possible.

Furthermore, despite the higher cognitive representation among silicosis patients, patients continue to be exposed to dust underestimating the risk of silicosis because they had unrealistic optimism. Finally, the study also provides a rationale for developing interventions that alter the illness perceptions of silicosis patients to promote and change unrealistic optimism to functional optimism that positively affects overall HRQoL. Additionally, the implications of the study for nursing education, practice, and research have been discussed.
